# Postural alterations and pulmonary function of mouth-breathing children

**DOI:** 10.1590/S1808-86942010000600002

**Published:** 2015-10-19

**Authors:** Waleska da Silveira, Fernanda Carvalho de Queiroz Mello, Fernando Silva Guimarães, Sara Lucia Siveira de Menezes

**Affiliations:** 1 MSc; Assistant Professor - Medical School of the Federal University of Rio de Janeiro; 2 Post-doctor; adjunct professor - Medical School of the Federal University of Rio de Janeiro; 3 PhD; Adjunct Professor - Medical School of the Federal University of Rio de Janeiro; 4 PhD; Adjunct Professor - Medical School of the Federal University of Rio de Janeiro. Graduate Program in Clinical Practice - Medical School of the Federal University of Rio de Janeiro

**Keywords:** posture, spirometry, postural balance, mouth breathing

## Abstract

Mouth-breathing children have changes in their stomatognathic system, which result in head projection, stress increase in the scapular belt muscles and postural adaptations. Although thoracic shape and posture can influence ventilatory dynamics, we didn't find studies addressing pulmonary function of mouth-breathing children.

**Aims:**

this study aimed at analyzing the posture of mouth-breathing children, and studying the existence of correlations between posture and pulmonary volumes.

**Material and Methods:**

prospective, observational and cross-sectional study, where the posture and pulmonary function of 17 mouth-breathing children and of 17 nasal-breathing children were evaluated by means of photogrammetry and forced spirometry.

**Results:**

when compared to nasal-breathing, mouth-breathing subjects presented an increment in head projection and cervical lordosis, forwarded gravity center and reduced pulmonary volumes. There was an association between head projection and forced vital capacity, and between postural alterations and age.

**Conclusion:**

mouth-breathing children have postural alterations which increases with age and also reduced spirometry values. The vital capacity reduction correlates negatively with head projection.

## INTRODUCTION

Mouth breathing is a disorder seen among schoolage children, of multifactorial etiology, causing morphological changes to the stomatognathic system as well as to posture. Mouth breathing in children causes mainly nasal disorders, allergic rhinitis or adenoid enlargement^1^, changes to maxilla development, changes to tongue position and mandible, influencing postural adjustment^2^. In order to facilitate air passage through the mouth, the patients project the head forward, increasing neck lordosis and shortening the sternocleidomastoid, scalene and chest musclues^3^, ^4^. Since the posture muscles act in synergism and to keep balance centered and thus, posture balance, such changes cause shoulder protrusion and scapular elevation^5^, kyphosis, increase in lumbar lordosis and anterior projection of the pelvis^6^. During ventilation these postural changes cause a more apical ventilatory pattern, changing chest-abdomen dynamics, which could reduce the diaphragm apposition zone^6^. Although respiratory muscle dysfunction and posture disorders are factors which determine ventilatory disorders, we did not find studies which characterize the ventilatory function in mouth breathing children and their association with postural changes, as well as postural changes and age. This study aimed at assessing postural changes based on age, as well as their association with the respiratory function in mouth breathing children.

## MATERIALS AND METHODS

This is an observational, cross-sectional study. The project was approved by the Ethics in Research Committee of the Clementino Fraga Filho University Hospital (HUCFF) of the Federal University of Rio de Janeiro (UFRJ), under protocol # 043/2004. Those responsible for the children who participated in the study signed the “Free and Informed Consent Form “, according to resolution 196/96 from the CNS/CONEP.

In the study we included nasal breathing (NB Group) or mouth breathing (MB Group) children with ages between eight (8) and twelve (12) years. After diagnostic confirmation, the children from the MB group were referred to the Clinical Pediatrics Ward of the Instituto de Puericultura e Pediatria Martagão Gesteira (UFRJ) and the Dentistry School of the UFRJ. The children from the NB group were recruited at the Tia Ciata Municipal School, located at Praça Onze, Rio de Janeiro. Exclusion criteria for both groups were: physical therapy, diagnosis or history of acute or chronic respiratory disease; neurofunctional, osteomuscular or cognitive dysfunction.

The postural analysis was carried out by a single examiner using the photogrammetric method^3^, in a reserved environment and located at the Physical Therapy Ward of the HUCFF. Before being photographed, 34 children were prepared with orange-color adhesives, placed on the following anatomical points: glabella; right and left temporomandibular joint midline; right and left acromyo-clavicular joint. Neck and back lordosis were marked with a cylindrical object of known length, placed by the examiner at the deepest points of the aforementioned landmarks (according to a palpatory assessment). The photographs were done using three Mirage® (Multilaser Industrial LTDA, SP, Brazil) tripods, 3 meters away from a blue background, in order to photograph on the anteroposterior (AP), posteroanterior (PA) and right side (D) positions. We used a 3.13 megapixel camera (Yashica - Brazil) with focus and image adjusted on the umbilicus line of the individual. The images were transferred to a matching computer and analyzed by the Fisiometer 3.0^®^ (Fisiometer Ltda, RJ, Brazil) software, previously validated^7^. An 11cm ruler was fixed to the child's body, to serve as a metric reference in order to adjust the software scale.

The head projection (PC), shoulder projection (PO), neck lordosis (LC) and lumbar lordosis (LL), were evaluated in the sagittal plane, with measurements using a trace from the background line to the markings on the temporomandibular joint, the acromyo-clavicular joint and deeper points of the lumbar and neck lordosis. We also analyzed the body positions in relation to the center of gravity (CG), classifying them as: normal, anterior or posterior.

The pulmonary function was assessed by means of forced spirometry, using the Easy One^®^ (Model 2001, ndd Medizintechnik AG, Zurich, Switzerland) spirometer, certified by the *American Thoracic Society* (ATS). The tests were carried out according to the ATS guidelines^8^ and those from the Brazilian Association of Pneumology^9^, and the following parameters were analyzed: forced vital capacity (FVC); forced expiratory volume in the first minute (FEV_1_) and FEV_1_/FVC ratio.

The statistical analysis was carried out with the SPSS 11.0 software (SPSS Inc., Chicago, IL), by utilizing the Mann-Witney and Spearmann's correlation tests. The significance level was set up in 5%.

## RESULTS

**Figure 1:**
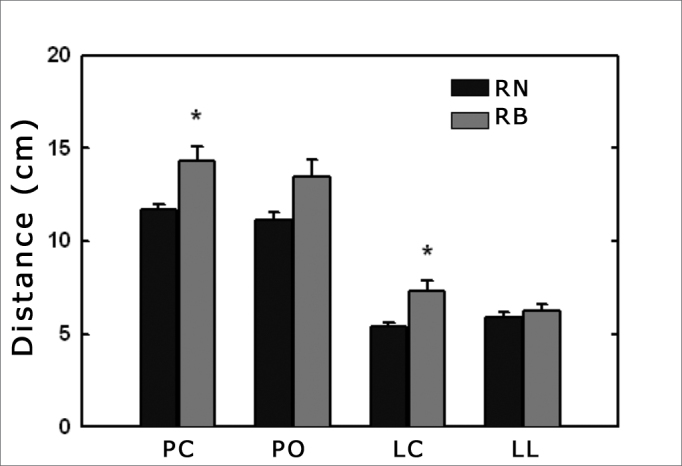
Measuring the distances of the posture variables in relation to the posterior plane. NB = group of nasal breathing children; MB = mouth-breathing children; PC = head projection; PO = shoulder projection; LC = Neck lordosis; LL =Lumbar lordosis. * significantly different in relation to NB (p<0.05).

We assessed 17 nasal breathing children and 17 mouth breathing ones. The group of nasal breathers was made up of 9 girls and 11 boys, with mean age of 8.6 years, while the group of mouth breathers was made up of 7 girls and 10 boys, with mean age of 8 years, and there was no statistically significant difference between the groups. We observed greater distances in relation to the back line in the MB group when compared to the variables: PC (14.3 vs. 11.7 cm; p = 0.005) and LC (7.3 vs. 5.4 cm; p = 0.016). There was no difference in the following variables: PO (13.5 vs. 11.1 cm; p = 0.2) and LL (6.3 vs. 5.9 cm; p = 0.49) between the groups. The results of the postural analysis for the NB and MB groups can be seen on Figure 1.

**Figure 2:**
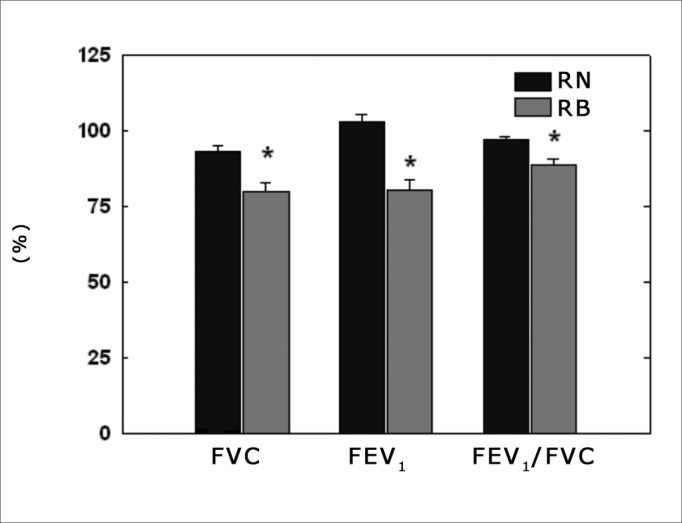
Spirometric variables. NB = nasal-breathing children; MB = mouth-breathing children; CVF = forced vital capacity; FEV1 = forced expiratory volume in the first second; * significantly different in relation to NB (p< 0.05).

As far as spirometry is concerned, all the variables analyzed had estimated values lower in the MB group when compared to their NB counterparts: CVF (79.8 vs. 93.3 %; p = 0.003), FEV_1_ (80.3 vs. 103.1 %; p = 0.0000004), FEV_1_/CVF (100.8 vs. 110.4 %; p = 0.000006). These results can be seen on Figure 2.

Concerning the association between variables, significant results between postural changes and age were obtained from group MB (Table 1). In the NB group we observed a positive correlation only between age and LL. The head projection was positively correlated with the forced vital capacity in the MB group (r = 0.5; p = 0.03).

**Table 1:** Correlation between posture and age.

	PC x age		PO x age		LC x age		LL x age	
	r	p	r	p	r	p	r	p
NB	0,01	0,95	0,36	0,15	-0,05	0,83	0,65	0,005*
MB	0,55	0,02*	0,65	0,004*	0,64	0,005*	0,61	0,009*

NB = nasal-breathing group; MB = mouth-breathing group; PC = head position; PO = shoulder projection; LC = neck lordosis; LL = lumbar lordosis.

* value < 0.05.

## DISCUSSION

Our results are in agreement with those from other authors who described head and shoulders projection forward in mouth breathing children^3^, ^5^, ^10^, ^11^. These findings are coherent with the forward shifting of the center of gravity, observed in 70% of the children from the MB group in our study, while in the NB group, most of the children (70 %) had normal center of gravity. The head projection and the increase in neck lordosis seen in mouth breathers is justified by the fact that these children commonly have changes in their stomatognathic system which, in fact, increases the tension on the head and shoulder muscles, changing their position in the anteroposterior or lateral directions^10^, ^11^, ^12^. In our study we did not measure the lateral head tilt because of the lack of validated reference points which would enable such assessment .

Lumbar lordosis and shoulder projection did not show significant differences between the groups; nonetheless this is one expected result, since this type of asymmetry is common to the age range investigated^13^.

Spirometry evaluations showed a significant reduction in the pulmonary values for MB group in relation to the NB. These results are similar to those found by Barbiero et al.^14^, who found a reduction in the forced vital capacity in functional mouth breathers, characterizing a restrictive pattern. It is very likely that the shortening of the scapular waist complex muscles, as well as diaphragmatic dyskinesia were determinant of these changes^6^. This statement is corroborated by the negative correlation between forced vital capacity and head projection observed in the MB group. Paradoxically speaking, head projection aims at facilitating air inflow through the mouth, ending up resulting in posture changes which would determine a worsening in pulmonary function. These changes tend to progress with the passing of the years, as per observed in our results: with an increase in age, posture adaptations intensify in order to make up for the drop in vital capacity, causing a progressive increase in head projection and neck lordosis (Table 1).

Numerous studies have assessed the association between respiratory dysfunction and posture changes in other clinical situations; nonetheless, in such papers muscle-skeletal changes and posture balance changes are seen as consequence of the additional stress employed during normal ventilation^15^, ^16^, ^17^. The results from our study bring about an important contribution as they show changes to the respiratory function arising from posture changes. Thus, we argue that the posture changes seen in patients with respiratory diseases may contribute to a worsening in pulmonary function, creating a feedback system which generates a progressive worsening from the respiratory and muscle-skeletal viewpoint.

Our study has shown that mouth breathing changes the stomatognathic system, altering posture and pulmonary function, thus signaling the need for a multidisciplinary approach for mouth-breathing children. In these regards, special attention must be given to head projection and neck lordosis increase, since a change in head position impacts the balance of muscle chains, triggering respiratory and posture adaptations.

## CONCLUSION

Mouth-breathing children have head projection and neck hyperlordosis which increase with age, besides reduction in spirometry values. Vital capacity reduction is negatively correlated with head projection.
